# A Case of *Trueperella bernardiae* Bacteremia due to a PICC-Associated Infection in a Paraplegic Patient

**DOI:** 10.1155/2023/6238339

**Published:** 2023-12-04

**Authors:** Carter Chapman, Jacob Nichols

**Affiliations:** Department of Internal Medicine, Infectious Disease Division, Texas Tech University Health Sciences Center School of Medicine, 3601 4th St, Lubbock, TX 79430, USA

## Abstract

*Trueperella bernardiae* is a Gram-positive bacterium known to cause a wide variety of opportunistic infections in humans. We report a novel case of *T. bernardiae* bacteremia in a paraplegic patient due to a peripherally inserted central catheter- (PICC-) associated infection that was treated successfully with piperacillin/tazobactam.

## 1. Introduction

**Figure 1:**
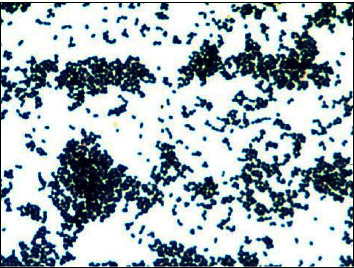
*Trueperella bernardiae* is a Gram-positive coccobacillus that is non-spore-forming and non-motile. The organism can appear singly, in clusters, or in pairs [1].

*Trueperella bernardiae* is a Gram-positive coccobacillus that is non-spore-forming and non-motile. The organism can appear singly, in clusters, or in pairs and is a facultative anaerobe (Figure 1) [1, 2]. Biochemically, it is both catalase and oxidase negative and demonstrates variability in its hemolytic activity. It was previously taxonomically placed in the genus *Actinomyces* and then reclassified within the genus *Arcanobacterium* before being placed in the genus *Trueperella* in 2011 [2]. *T. bernardiae* is known to cause infections in humans, most commonly presenting as wound infections in diabetic or postsurgical patients [3]. We present a case of *T. bernardiae* bacteremia due to a PICC-associated infection in a paraplegic patient.

## 2. Case Presentation

A 45-year-old male with a past medical history of T4-T5 spinal cord injury in 1994 and multiple hospitalizations for infections requiring extended courses of antibiotics was admitted for severe muscle spasms, abdominal pain, nausea, and vomiting. Labs on admission demonstrated neutrophilic leukocytosis and high anion gap metabolic acidosis secondary to lactic acidosis. A computed tomography (CT) scan of the abdomen and pelvis revealed a left hip joint fluid collection and appendiceal wall thickening concerning for appendicitis. Blood cultures were obtained, and empiric antibiotic therapy was initiated with intravenous (IV) cefepime and vancomycin. The patient was evaluated by the surgical team and felt to not have acute appendicitis. The patient then underwent left hip arthrocentesis with return of 3 mL of bloody, cloudy synovial fluid. Due to the small volume, fluid analysis was not performed and the sample was sent for Gram stain and culture which were negative. Blood cultures also revealed no growth. IV cefepime and IV vancomycin were continued for eight days until the patient was discharged, and upon discharge he was transitioned to IV cefepime and IV daptomycin through a PICC line (Bard, double lumen, 5 French catheter) for 4 weeks as an outpatient. Both according to our electronic medical records and outside medical records that we have received, there is no previous documentation of PICC insertion in this patient. Unfortunately, the patient did not show for his hospital follow-up in the infectious disease clinic.

Three months later, the patient was readmitted due to concerns for another episode of septic arthritis in his left hip. Upon admission he was noted to still have his left upper extremity PICC from his previous hospitalization, indicating that the PICC was in place for approximately three months, about eight weeks longer than it was supposed to be in place. The PICC remained in place during this time because the patient was lost to follow-up after his previous discharge and did not show for his follow-up appointment in the infectious disease clinic and was therefore unable to have it removed. It is unclear if the patient's PICC was cared for during this time. Two sets of peripheral blood cultures were obtained, and the patient underwent left hip drain placement by interventional radiology from which cultures were negative. A superficial culture obtained from the patient's chronic left hip wound was reported as positive for normal flora consistent with the site. On hospital day 3, both sets of blood cultures became positive with reported growth of Gram-negative rods at which point the patient was started on IV piperacillin/tazobactam. A repeat pair of quantitative blood cultures was obtained which revealed growth of Gram-positive cocci in clusters 15 hours later. IV vancomycin was initiated, and the infectious disease service was consulted with the recommendation to continue current antibiotics, remove the PICC, and obtain repeat blood cultures. By this time, the Gram-negative rods had been identified as *Trueperella bernardiae* by matrix-assisted laser desorption/ionization time of flight (MALDI-TOF). It was suspected that the initial Gram stain had been over-decolorized resulting in the read of Gram-negative rods. *T. bernardiae* was not worked up further for antibiotic susceptibility, but a decision was made to continue piperacillin/tazobactam for antibiotic therapy based on review of the literature concerning *T. bernardiae.* The Gram-positive cocci from the repeat blood cultures were subsequently identified as methicillin-resistant *Staphylococcus aureus* (MRSA). Search for an alternative source of infection was negative, and the patient improved after removal of the PICC, so although no segmental cultures of the catheter body or definitive studies of the catheter were performed, the final diagnosis of central line-associated bloodstream infection from his PICC was made on a clinical basis. Repeat paired quantitative blood cultures obtained after PICC removal were both negative for growth at five days, after which the patient was discharged on IV piperacillin-tazobactam for two weeks for the *T. bernardiae* bacteremia and IV daptomycin for four weeks for his MRSA bacteremia, both through a tunneled PICC line. The tunneled PICC line was subsequently removed after completion of this regimen.

## 3. Discussion

In this case, treatment of *T. bernardiae* consisted of piperacillin/tazobactam 13.5 g IV q24 h via continuous infusion for total of 2 weeks. This decision was made based on case reports of the organism's susceptibility to this antibiotic, and it would also provide adequate coverage of a suspected complicated skin and skin structure infection of the patient's left hip. Previous case reports have shown that *T. bernardiae* is also susceptible to linezolid, moxifloxacin, vancomycin, and rifampicin. Although recent advances in microbiological detection, such as MALDI-TOF, have increased the incidence of detection and the subsequent treatment of *T. bernardiae* infections, it remains susceptible to most antibiotics at this time, with resistance to only aminoglycosides, penicillin G, and metronidazole reported [4, 5].

An extensive literature review reveals cases of *T. bernardiae* infection in several countries around the world. However, these cases are mostly reports on *T. bernardiae* wound infections—either secondary to surgical procedures or diabetic foot ulcers. There are also a limited number of reported cases that do not describe wound infections and involve, namely, septic thrombophlebitis in an injection drug user, a brain abscess in a patient with chronic otitis media, a breast abscess, and a case of bacteremia that presented with sepsis secondary to pelvis osteomyelitis, micro-abscesses, and septic arthritis [3, 5]. To our knowledge, this is the first case of *T. bernardiae* bacteremia due to infection of a PICC line. The infection was likely due to the PICC line remaining in place after completion of previous intravenous antibiotic therapy.

Peripherally inserted central catheters, or PICCs, are a form of central venous access used for intermediate to long-term use, with average time in situ ranging from one week to six months [6]. While PICCs can allow for the outpatient treatment of bacteremia, septic arthritis, or osteomyelitis, as well as the administration of chemotherapeutic drugs, they are associated with significant complications, including central line-associated bloodstream infections and deep venous thrombosis [7, 8]. The use of PICCs has increased in recent years, so has the incidence of these complications [9, 10]. Because of the morbidity and mortality associated with these complications, it is essential that all central venous catheters (CVCs), including PICCs, are properly managed. This includes avoiding unnecessary catheterization, removing CVCs that are no longer necessary, ensuring proper nurse, physician, and patient education concerning CVCs and their complications, and maintenance of sterile techniques when administering medications and tending to the catheter [11].

This case serves to add to the growing body of reports of *T. bernardiae* infection in humans by presenting a novel infection source and patient population affected by this organism. In addition, it serves to corroborate the use of piperacillin/tazobactam for *T. bernardiae* described in previous case reports given our successful use in this case. Finally, our case highlights the importance of proper management, hygiene, and patient education about PICCs.

## 4. Conclusion

*Trueperella bernardiae* is a Gram-positive opportunistic pathogen known to cause infections in primarily postsurgical and diabetic patients; it has also been reported as the causative agent in a variety of other infections, including septic thrombophlebitis and septic arthritis. Despite increases in its detection as a pathogenic organism—due to the advent and implementation of MALDI-TOF—and resultant antibiotic therapy, it remains sensitive to most antibiotics at this time [5]. This case report describes a case of *T. bernardiae* bacteremia in a paraplegic patient secondary to a PICC-associated infection, the first to our knowledge to be reported in the literature. This case also serves to add to the extensive body of literature concerning the use and complications of central venous catheters, including PICCs, in the realm of infectious disease.

## Data Availability

The data used to support the findings of this study are included within the article.
